# Awareness of isotretinoin use and safety in Saudi Arabia: A nationwide cross-sectional study

**DOI:** 10.1016/j.jsps.2023.101796

**Published:** 2023-09-26

**Authors:** Abdullah A Alshehri, Bander A Althobaiti, Wael Y Khawagi, Kevin D Murphy

**Affiliations:** a Department of Clinical Pharmacy, College of Pharmacy, Taif University, Al Huwaya, Taif 26571, Saudi Arabia; b Pharmaceutical Care Research Group, School of Pharmacy, University College Cork, Cork, Ireland; c King Abduaziz Specialised Hospital, Ministry of Health, Taif, Saudi Arabia

**Keywords:** Medication Safety, Awareness, Isotretinoin, Saudi Arabia, Cross-sectional study

## Abstract

**Introduction:**

Acne is a prevalent skin condition that affects numerous adolescents and adults worldwide. The most effective treatment for acne is isotretinoin, but its usage is associated with a wide range of adverse effects, and regular monitoring is necessary. Hence, appropriate usage with awareness of potential side effects is crucial. This study aimed to assess the knowledge and awareness of isotretinoin use and safety among individuals with acne in Saudi Arabia.

**Methods:**

A national cross-sectional survey was conducted through an online self-administered questionnaire distributed via social media platforms. The questionnaire consisted of 27 questions in multiple-choice and Likert scale formats, covering demographics, patient awareness of isotretinoin use and side effects, satisfaction with clinical consultation and information provided. Descriptive statistics were used to summarize data.

**Results:**

1315 participants completed the survey, of which most were female (74.1%), single (67.5%), and aged 18–25 years (48.9%). Dryness and teratogenicity were the most commonly known side effects of isotretinoin use (85.5% and 64.9% respectively). However, most participants were unaware of other side effects, such as psychiatric disorders (62.9%), altered liver enzyme concentrations (65.2%), hyperlipidemia (68.1%), anemia (92.4%), and decreased platelet count (96%). Moreover, 36% of sexually active females initiated isotretinoin without contraception. Regarding satisfaction with the information provided during clinical consultation, 63.2% of participants were very satisfied or satisfied. Doctors were the primary source of information (86.8%), followed by the internet (17.8%). Only 45% were informed to avoid blood donation during and after treatment for at least two months.

**Conclusion:**

The study highlights the significance of providing patients with comprehensive information about the potential side effects of isotretinoin, including the need to use contraception and avoid blood donation during and after treatment. Effective communication between physicians and patients is critical in ensuring the safe and effective use of isotretinoin.

## 1 Introduction

Acne vulgaris (AV) is a prevalent dermatologic condition affecting people of all ages and genders worldwide, with a reported prevalence of up to 85% among adolescents (Bhate and Williams, 2013). In Saudi Arabia, the exact prevalence of acne is not well-established, but a few studies have reported a high prevalence of acne among adolescents and young adults, ranging from 56% to 71% (Al Robaee, 2005, Bajawi et al., 2016, Abo El-Fetoh et al., 2016, Bahattab et al., 2017). AV can have a significant impact on patients' quality of life, causing not only physical discomfort and scarring but also psychological distress.

While acne is often treated with topical agents or antibiotics, most cases may require oral systemic isotretinoin (*13-cis-retinoic acid*) (Layton, 2009, Wiegand and Chou, 1998). Although isotretinoin is widely recognized as an effective medication for treating AV (Eichenfield et al., 2021, Zaenglein et al., 2016), it is also associated with a range of potential side effects, including teratogenicity, psychological disorders, dryness, hyperlipidemia and depression (McLane, 2001, Brzezinski et al., 2017, Charakida et al., 2004). The risk of these side effects highlights the importance of patient awareness and adherence to guidelines and recommendations for safe and effective treatment (Lee et al., 2016, Alanazi, 2020, Al-Harbi, 2010).

Although some studies have been conducted in several countries regarding the awareness of isotretinoin use and safety, in Saudi Arabia, research has been limited to specific populations or regions. To date, two studies assessing the knowledge and use of isotretinoin has focused only on female college students (Albadr et al., 2019, Bakheet et al., 2020). Three studies evaluated the awareness of using isotretinoin and it is side effects in specific regions Al-Madinah (Molla et al., 2020), Alahsa (Younis and Al-Harbi, 2019) and Al-Qassim region (Al-Harbi, 2010). However, the findings of these studies may not be generalizable to the entire country, where established services and funding bodies are different. Given the widespread use of isotretinoin in Saudi Arabia and the potential risks associated with its use, there is a need to assess patients’ awareness and understanding of isotretinoin use and safety across the country. Therefore, it is essential to obtain a comprehensive understanding of patients’ awareness of isotretinoin use and safety among patients with AV across Saudi Arabia including isotretinoin usage and side effects, and patients’ satisfaction of their consultation and information given in clinic such as routine blood tests requested prior to administration and recommendations to ensure the safe and effective use of isotretinoin and improve patient education.

## 2 Methods

### 2.1 Study design

A national cross-sectional survey was conducted using an online self-administered questionnaire was conducted via social media platforms for a period of four months between May and August 2022. Adults with acne who had used isotretinoin as a treatment and lived in Saudi Arabia were eligible to participate. Participation in the study was voluntary, and data was subjected to strict protocols to ensure confidentiality.

### 2.2 Questionnaire development

The questionnaire was developed following a previous validated survey tool and discussion with four academic pharmacists (Molla et al., 2020, Younis and Al-Harbi, 2019). The online questionnaire was designed using SurveySparrow, and prefaced with an introduction including the study title, the study’s aim and objectives, data confidentiality information, researchers’ contact information; and a statement of consent to participate in the study by ticking a check box. The survey consisted of 27 questions in a multiple-choice and 5-point Likert scale formats. The questionnaire included three major domains: (1) demographics such as gender, age, civil status and region of living, (2) Patients’ awareness of isotretinoin use and its side effects, and (3) Patients’ satisfaction of their consultation and information given in clinic. The questionnaire was designed in attractive colors and illustrative pictures to reduce the potential for double responses. The questionnaire was translated into Arabic, checked by two independent bilingual academics, and validated via pilot study. The questionnaire was expected to take around 10 min to complete.

### 2.3 Piloting the questionnaire

The questionnaire was piloted by seven randomly selected participants to verify the readability, understanding, and feasibility of the online questionnaire. Based on their feedback, one irrelevant question was removed and some questions were amended to use lay terms. Participants in the pilot phase were excluded from the analysis to ensure that their responses did not bias the results of the study.

### 2.4 Data collection

The online questionnaire was distributed through a link to social media platforms (Twitter, LinkedIn and WhatsApp). The study link and flyer was sent in the same attractive colors to avoid duplication. Data collection was conducted for a period four months between May and August 2022.

### 2.5 Data analysis

The data from the questionnaire was analyzed using statistical package SPSS (IBM SPSS Statistics 29) and the analysis randomly double-checked by another member of the research team. Descriptive statistics were used to summarize the data. A Chi-square test was performed to identify associations between demographic factors, such as age, treatment duration, and the severity of the condition, with awareness levels. Statistical significance was assessed at *p* < 0.05, and all tests were two-tailed.

## 3 Results

### 3.1 Characteristics of respondents (*n* = 1315)

A total of 1315 patients completed the online survey. There were 975 female participants (74.1%), 67.5% were single, and 48.9% of them were aged 18–25 years. Regarding the severity of the condition, the majority of participants (94%) had mild to moderate acne. About 40% of participants reported that they were taking isotretinoin for about 6–8 months. Most of participants (72.2%) had received their prescriptions from a private clinic. One third of them lived in Makkah, followed by 12.5% and 12.3% who lived in Al-Madinah and Riyadh, respectively. Table 1 shows the characteristics of the participants.

**Table 1:** Demographic characteristics of respondents.

Characteristics		Number (%)
Gender		
	Female	975 (74.1)
	Male	340 (25.9)
Civil status		
	Single	887 (67.5)
	Married	428 (32.5)
Age group (year)		
	18–25	643 (48.9)
	26–35	518 (39.4)
	36–45	139 (10.6)
	46–55	15 (1.1)
Severity of the condition		
	Mild	582 (44.3)
	Moderate	650 (49.4)
	Severe	82 (6.2)
Duration of using treatment		
	1–2 months	215 (16.3)
	3–5 months	464 (35.3)
	6–8 months	503 (38.3)
	9–12 months	133 (10.1)
Type of clinic where medication was prescribed		
	Public	366 (27.8)
	Private	949 (72.2)
Regions		
	Makkah	397 (30.2)
	Riyadh	165 (12.5)
	Al-Madinah	162 (12.3)
	Najran	129 (9.8)
	Asir	96 (7.3)
	Tabuk	86 (6.5)
	Al-Qasim	54 (4.1)
	Hail	53 (4.0)
	Western region	52 (4.0)
	Jazan	46 (3.5)
	Al-Bahah	41 (3.1)
	North region	22 (1.7)
	Al-Jouf	12 (0.9)

### 3.2 Awareness of isotretinoin use and its side effects

**Fig. 1:**
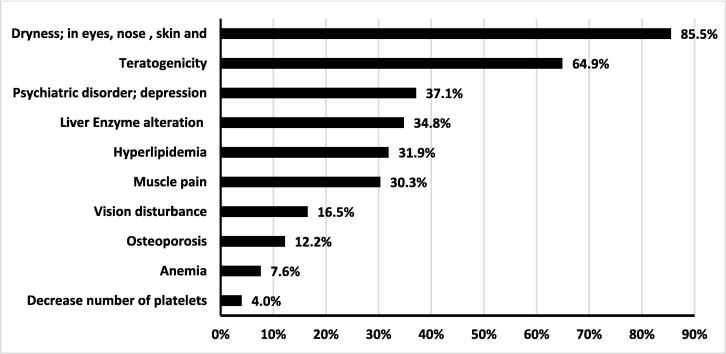
Percentage of participants awareness of side effects.

When considering the side effects associated with the use of isotretinoin, few effects were relatively well-known among participants. Most participants (85.5%) were aware of dryness in the eyes, nose, and skin, and 64.9% were aware of the teratogenicity of isotretinoin. However, there were several other side effects that more than half of participants were not familiar with. For instance, 62.9% of participants were unaware that isotretinoin could be associated with psychiatric disorders such as depression and anxiety. Similarly, altered liver enzyme concentrations were unknown to 65.2% of participants. Hyperlipidemia was not recognized as a potential side effect by 68.1% of participants. Also, 92.4% of participants were unaware of the risk of anemia, and an overwhelming 96% were not aware that isotretinoin could lead to a decreased platelet count [Fig. 1].

Out of 975 female participants in the survey, 324 (33%) were married and 282 (29%) were sexually active and able to get pregnant. Among these 282 participants, 277 (98.2%) were informed of the contraindication of isotretinoin during pregnancy and 218 (77.5%) had performed two pregnancy tests before starting isotretinoin. However, 101 (36.1%) sexually active females initiated isotretinoin without contraception, while 181 (63.9%) participants reported being advised to use contraception. Of those who were advised to use contraceptives, 127 (69.1%) were advised to use only one method of contraception whereas 54 (29.9%) used two methods. Furthermore, about 255 (90.7%) reported being aware of the need for an interval between the end of treatment and the start of attempts to conceive [Table 2].

**Table 2:** Perceptions and practices regarding isotretinoin usage among married fertile females.

Statement		Number (%)
	Is the participant sexually active and able to get pregnant? (n = 324)	
	Yes	282 (86.7)
	No	43 (13.2)
Patient informed that isotretinoin is contraindicated during pregnancy (n = 282)		
	Yes	277 (98.2)
	No	5 (1.7)
Patients performed two pregnancy tests prior to initiating isotretinoin (n = 282)		
	Yes	218 (77.5)
	No	64 (22.5)
Patients advised to use any contraception (n = 282)		
	Yes	181 (63.9)
	No	101 (36.1)
Number of Method/methods of contraception where you advised to use (n = 181)		
	1	127 (69.1)
	2	54 (29.9)
Informed that if wanted to conceive, isotretinoin must be discontinued at least 6 months prior to attempts to conceive (n = 282)		
	Yes	255 (90.7)
	No	27 (9.3)

### 3.3 Patients’ satisfaction of their consultation and information given

**Fig. 2:**
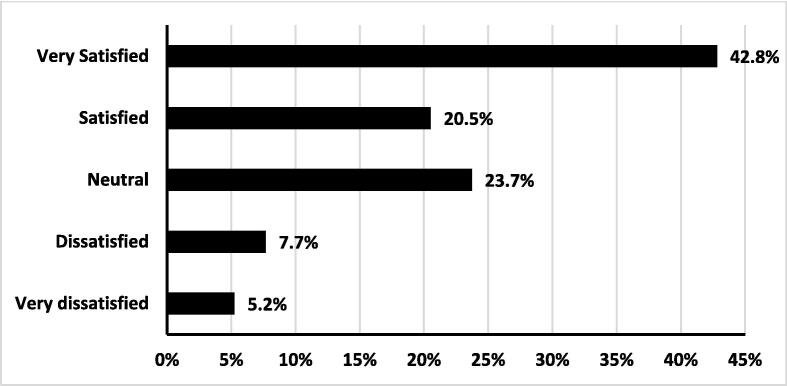
Satisfaction with the provided information in the consultation.

The results regarding participants' satisfaction with the information given about isotretinoin during their clinical consultation revealed that most respondents, 63.2%, reported being either very satisfied or satisfied with the information provided. Conversely, only a small percentage, 12.9%, expressed being either very dissatisfied or dissatisfied with the information they received [Fig. 2].

Before prescribing isotretinoin, physicians requested routine blood tests including lipid profile measurements for 1125 patients (85.6%), liver enzymes for 1137 patients (86.5%), and glucose levels for 903 patients (68.7%). Most participants were informed to avoid direct exposure to sunlight (65.2%) and to use sunlight protection while on isotretinoin. Results demonstrate that about 45% of participants had been informed that blood donation should be avoided during the treatment and for at least two months after the treatment has been discontinued [Table 3].

**Table 3:** Patients’ perception regarding physicians’ practices prior to prescribing isotretinoin.

Variable		Number (%)
Physician took a blood sample to measure the lipid profile before prescribing isotretinoin		
	Yes	1125 (85.6)
	No	190 (14.4)
Physician took a blood sample to measure the liver enzyme before prescribing isotretinoin		
	Yes	1137 (86.5)
	No	178 (13.5)
Physician took a blood sample to measure the glucose level before prescribing isotretinoin		
	Yes	903 (68.7)
	No	412 (31.3)
Patients informed to avoid the direct exposure to the sunlight		
	Yes	857 (65.2)
	No	458 (34.8)
Patients informed to use sunlight protection while on isotretinoin?		
	Yes	921 (70.0)
	No	394 (30.0)
Patients informed not to share your medication (isotretinoin) with others		
	Yes	1180 (89.7)
	No	135 (10.3)
Patients tried other topical treatment prior to initiating isotretinoin		
	Yes	661 (50.3)
	No	653 (49.7)
Patients informed to not donate blood until at least two months after the last dose		
	Yes	611 (46.5)
	No	704 (53.5)

**Fig. 3:**
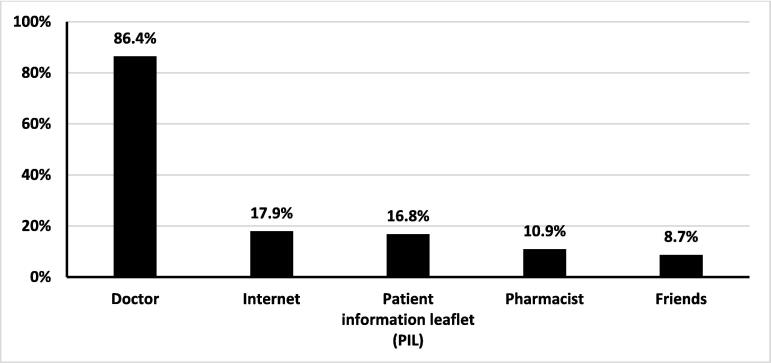
Drug information sources.

The findings regarding the resources used by participants to obtain information about isotretinoin demonstrated that many participants relied on multiple sources. The primary source of information for participants was doctors, with 86.8% reporting that they obtained information from healthcare professionals. The internet was the second most common source, utilized by 17.8% of participants. Patient information leaflets also played a role in providing information [Fig. 3].

## 4 Discussion

To the author’s knowledge, this is the first national cross-sectional study in Saudi Arabia to assess patients’ awareness and perception of isotretinoin use and safety, including their views on medication side effects and satisfaction with clinical consultations and information provided. The majority of participants were young females with mild to moderate acne who had been taking isotretinoin for 6–8 months. The study findings indicate that dryness and teratogenicity were the most commonly known side effects of isotretinoin across Saudi Arabia. Most patients were advised to avoid direct sunlight and to use sunscreen while on isotretinoin. However, patient were not well-aware of other significant side effects such as psychiatric disorders, altered liver enzyme concentrations, hyperlipidemia, anemia and decreased platelet count. Additionally, patients were not adequately informed about the need to avoid blood donation during and after treatment for at least two months. This study highlighted concerns about the prescribing of a teratogenic medication to sexually active females without initiating contraception. Participants' satisfaction with the information provided during clinical consultations regarding isotretinoin was below average, with doctors being the primary source of information.

### 4.1 Comparison with existing literature

This study findings indicate that while participants demonstrated awareness of some side effects of isotretinoin, such as dryness, there was considerable lack of recognition of many common side effects such as psychiatric disorders and hepatic enzyme alteration. These findings are consistent with earlier studies conducted in Alahsa and Al-Qassim region that have reported variable levels of patient awareness of isotretinoin's side effects (Younis and Al-Harbi, 2019, Al-Harbi, 2010). One of the most serious adverse effects of isotretinoin is that it is potential to affect the patient's mental health. According to the responses, 63% of participants were unaware of isotretinoin's potential impact on mental state and its contraindication for patients with depression. Facial acne patients, particularly female patients with a longer treatment duration, are more likely to experience depression (Sood et al., 2020, Kang et al., 2015). As the severity of AV is positively associated with the intensity of depression, psychological care should be considered to improve treatment outcomes and quality of life.

Consistent with the findings of this study, a previous study conducted among college students in Riyadh reported incidents of pregnancy during isotretinoin treatment, highlighting the need for a pregnancy protection program for patients taking this medication (Albadr et al., 2019). Our study also highlighted issues related to sexually active females not initiating contraception, which further emphasizes the importance of healthcare providers ensuring that patients are aware of the potential risks of isotretinoin during pregnancy and taking appropriate measures to prevent pregnancy during treatment. Moreover, the study's findings demonstrate that doctors are the primary source of information on isotretinoin therapy, as reported by approximately 3-in-5 participants (61.6%) followed by the internet and patients' information leaflets. These finding aligns with previous research conducted in Saudi Arabia and Jordan (Albadr et al., 2019, Jarab et al., 2022). Similarly, Bakheet et al. (2020) found that doctors were also the primary source of information for participants. These results stress the importance of effective communication between patients and physicians to ensure the safe and effective use of isotretinoin.

### 4.2 Strength and limitations

The study has several strengths, including a large sample size that is significantly higher than previous studies conducted in Saudi Arabia, and the use of an online questionnaire that facilitated ease of responding and maximized recruitment of potential participants. Additionally, the study was national study covered all regions across the country, providing a comprehensive understanding of patients' awareness and perception of isotretinoin use and safety. However, the study also has limitations, such as the potential for selection bias due to the use of an online questionnaire, which could have resulted in a non-representative sample. The reliance on self-reported data from participants may have also introduced recall bias or social desirability bias. Finally, the study did not assess the actual prescribing practices of physicians, which could be an important factor in ensuring the safe and effective use of the medication.

### 4.3 Implications for research and practice

The findings of this study have important implications for practitioners and policy makers in Saudi Arabia. The study highlights the importance of providing comprehensive information to patients about the common potential side effects of isotretinoin, with a particular emphasis on those that are less commonly recognized in the country such as psychiatric disorders. The study also emphasizes the need for greater attention to be dedicated to educating physicians to ensure the safe and effective use of isotretinoin, and to improve patient education as the primary source of information, particularly on recommendations such as avoiding blood donation during and after treatment and the importance of contraception in sexually active females for at least two forms of contraception. Healthcare providers in Saudi Arabia should take note of these findings and develop strategies to improve patient education and communication about isotretinoin therapy to ensure its safe and effective use.

## 5 Conclusion

This study sheds light on patients' awareness and perception of isotretinoin use and safety in Saudi Arabia. The findings emphasize the importance of providing patients with comprehensive information about the potential side effects of isotretinoin. The study also highlights the need for physicians to initiate contraception in sexually active females before prescribing a teratogenic medication. Furthermore, the study suggests that patients' satisfaction with the information provided during clinical consultations particularly the need to avoid blood donation during and after treatment. Overall, the study highlights the importance of effective communication between patients and physicians in ensuring safe and effective use of isotretinoin.

## Funding

None.

## Ethics approval and consent to participate

This study obtained the ethical approval from the research and studies department, Directorate of Health Affairs, Taif, Saudi Arabia (IRB Registration Number with KACST, KSA: HAP-02-T-067, Approval number: 462).

## Availability of data and material

The datasets used and analyzed during the current study are available from the corresponding author on reasonable request.

## Declaration of Competing Interest

The authors declare that they have no known competing financial interests or personal relationships that could have appeared to influence the work reported in this paper.
